# Late gadolinium enhancement imaging using spiral readouts at 3T

**DOI:** 10.1186/1532-429X-15-S1-E8

**Published:** 2013-01-30

**Authors:** Iain Pierce, Jennifer Keegan, Peter Drivas, Peter D Gatehouse, David N Firmin

**Affiliations:** 1 NHLI, Imperial College London, London, UK; 2 NIHR Royal Brompton Cardiovascular Biomedical Research Unit, Royal Brompton Hospital, London, UK

## Background

Breath-hold late gadolinium enhancement (LGE) imaging is commonly performed as a stack of 2D short-axis slices through the ventricles. Standard inversion recovery with Cartesian k-space coverage requires long acquisition windows of 140 - 200 ms which have potential to introduce artefacts due to longitudinal magnetisation recovery and blurring due to cardiac motion. Ghosting in the phase encoding direction from beat-to-beat signal variations and due to poor breath-holding may mimic enhancement and a phase swapped (PS) stack of slices is also acquired to confirm diagnosis, although this doubles the scan time.

Spiral data acquisition provides highly efficient coverage of k-space and would potentially allow a significant reduction of the acquisition window. This would reduce motion blurring and edge artefacts while at the same time, may eliminate the need for PS acquisitions. Its use for 2D LGE was investigated.

## Methods

An interleaved spiral readout sequence was designed to match the current clinically-used standard inversion-prepared segmented gradient-echo sequence in terms of spatial resolution (1.4 x 1.4 x 6 mm (reconstructed 0.7 x 0.7 x 6 mm)) and breath-hold duration (14 cardiac cycles with alternate R-wave gating). The sequence consisted of 12 interleaves (13 ms duration) with 2 acquired per cardiac cycle (flip angles 45° & 90°). The acquisition window was 53 ms compared to 144 - 204 ms for the standard sequence. A water excitation pulse eliminated signal from fat and associated off-resonance blurring.

Spiral LGE imaging was performed after the completion of clinical LGE studies (with and without PS) in 9 patients on a Siemens 3T Skyra scanner. Basal, mid and apical short axis slices were acquired for paired comparison with the standard Cartesian images in terms of blood signal-to-noise ratio (SNR) and blood/myocardium contrast-to-noise ratio (CNR).

## Results

**Figure 1:**
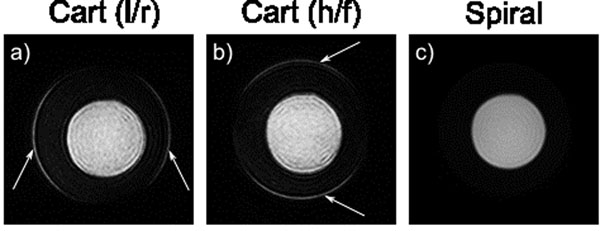
Images of a phantom using the standard LGE Cartesian sequence acquired using (a) left / right (l/r) and (b) head / foot (h/f) phase encoding and (c) when using the spiral sequence. The phantom consists of two circular bottles with one placed inside the other, mimicking the left ventricular blood pool and myocardium. The bottles were filled with different concentrations of gadolinium such that the inner bottle (LV blood pool) had a shorter T1 than the outer bottle (myocardium). The Cartesian LGE images ((a) & (b)) show edge artefact in the phase encoding direction, resulting from longitudinal magnetisation recovery through the long acquisition window (arrows). This artefact is not present on the LGE spiral image due to the much shorter acquisition window.

Figure 1 shows phantom scans with edge artefact using Cartesian k-space coverage (due to magnetisation recovery through the long acquisition window) which are not present on the spiral image.

**Figure 2:**
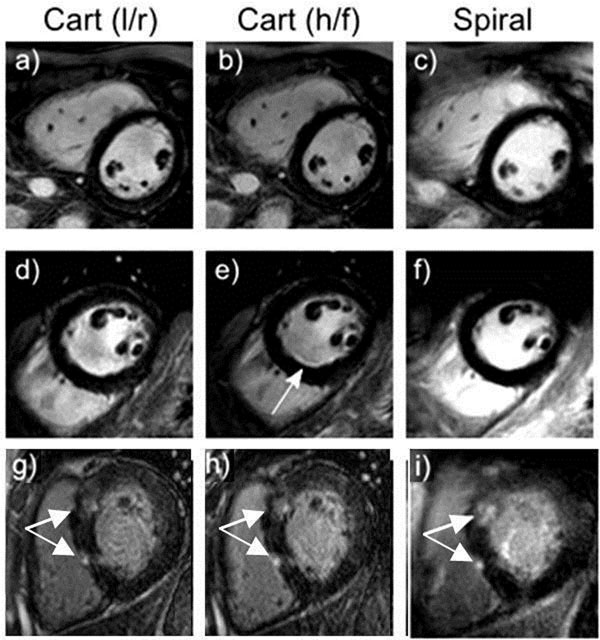
Example short axis images using standard Cartesian LGE with phase encoding in both the left/right (left column) (l/r) and head/foot (middle column) (h/f) directions together with the corresponding spiral LGE images (right column) for 3 example patients. The second patient (middle row) shows a bright edge artefact on (e) (arrow) which is not present on either (d) or (f). The third patient (bottom row) shows clearly delineated late enhancement (arrows) on the spiral image (i).

*In vivo*, blood pool SNR and blood-myocardium CNR were significantly higher with the spiral acquisitions (SNR: 162.8+/-62.5 vs 238.8+/-112.0, p<0.05, CNR: 137.8+/-55.8 vs 201.8+/-105.1, p < 0.05) despite always being acquired later when the blood pool gadolinium concentration is lower. Example images from both sequences in 3 patients are shown in Figure 2.

## Conclusions

Spiral readouts allow shorter acquisition windows then standard Cartesian LGE imaging (53 ms compared to 144-204 ms in this study) which has the potential to reduce edge artefacts and motion blurring. The higher flip angles used also result in increased SNR and CNR.

While beat to beat signal variations result in ghosting in the phase encode direction with Cartesian acquisitions, requiring the acquisition of a PS stack, their effect with spiral acquisitions is less obvious and will be a focus of future work.

## Funding

Wellcome Trust Grant WT093953MA

NIHR - National Institute for Health Research

